# Developmental Changes in Dendritic Spine Morphology in the Striatum and Their Alteration in an A53T α-Synuclein Transgenic Mouse Model of Parkinson’s Disease

**DOI:** 10.1523/ENEURO.0072-20.2020

**Published:** 2020-08-27

**Authors:** Laxmi Kumar Parajuli, Ken Wako, Suiki Maruo, Soichiro Kakuta, Tomoyuki Taguchi, Masashi Ikuno, Hodaka Yamakado, Ryosuke Takahashi, Masato Koike

**Affiliations:** 1 Department of Cell Biology and Neuroscience, Juntendo University Graduate School of Medicine, Tokyo 113-8421, Japan; 2 Department of Cellular and Molecular Neuropathology, Juntendo University Graduate School of Medicine, Tokyo 113-8421, Japan; 3 Laboratory of Morphology and Image Analysis, Research Support Center, Juntendo University Graduate School of Medicine, Tokyo 113-8421, Japan; 4 Department of Neurology, Kyoto University Graduate School of Medicine, Kyoto 606-8507, Japan; 5 Advanced Research Institute for Health Science, Juntendo University, Tokyo 113-8421, Japan

**Keywords:** α-synuclein, A53T, dendrite, dendritic spines, FIB/SEM, Parkinson’s disease

## Abstract

The aging process is accompanied by various neurophysiological changes, and the severity of neurodegenerative disorders such as Parkinson’s disease (PD) increases with aging. However, the precise neuroanatomical changes that accompany the aging process in both normal and pathologic conditions remain unknown. This is in part because there is a lack of high-resolution imaging tool that has the capacity to image a desired volume of neurons in a high-throughput and automated manner. In the present study, focused ion beam/scanning electron microscopy (FIB/SEM) was used to image striatal neuropil in both wild-type (WT) mice and an A53T bacterial artificial chromosome (BAC) human α-synuclein (A53T-BAC-*SNCA*) transgenic (Tg) mouse model of PD, at 1, 3, 6, and 22 months of age. We demonstrated that spine density gradually decreases, and average spine head volume gradually increases with age in WT mice, suggesting a homeostatic balance between spine head volume and spine density. However, this inverse relationship between spine head volume and spine density was not observed in A53T-BAC-*SNCA* Tg mice. Taken together, our data suggest that PD is accompanied by an abnormality in the mechanisms that control synapse growth and maturity.

## Significance Statement

Currently, the clinical diagnosis of Parkinson’s disease (PD) is based on the presence of motor symptoms. However, these symptoms only manifest after a significant proportion of dopaminergic cells have degenerated. The latent, prodromal phase is therefore of particular interest for the development of disease-modifying therapies to slow down or reverse the course of neurodegeneration. Although clinical markers to diagnose patients in the prodromal phase are gradually emerging, a structural marker that defines this phase has not yet been identified. Structural changes likely occur in the synapse before the onset of neurodegeneration. Thus, this study performed an ultrastructural analysis of synapses in a mouse model of prodromal PD and revealed distinct, age-dependent structural changes.

## Introduction

Parkinson’s disease (PD) is a progressive neurodegenerative disorder characterized by motor dysfunctions including rigidity, akinesia, tremor, and bradykinesia (Jankovic, 2008; Erro and Stamelou, 2017; Simon et al., 2020). Its pathologic hallmarks include the aggregation of α-synuclein in cellular inclusions, resulting in the formation of Lewy bodies, and the progressive loss of nigrostriatal dopaminergic neurons and consequent basal ganglia dysfunction (Polymeropoulos et al., 1997; Spillantini et al., 1998; Riederer et al., 2019; Tsukita et al., 2019). Currently, there is no effective cure for PD, and this is partly because of our lack of understanding about the precise synaptic-level changes that are associated with this disease. In particular, it remains unknown whether synaptic abnormalities precede the appearance of different behavioral phenotypes associated with PD. This knowledge may eventually aid in the early diagnosis of the disease.

Medium spiny neurons (MSNs), the principal neurons in the striatum, have dendrites that possess thorn-shaped dendritic spines. Because the vast majority of excitatory synaptic inputs impinge on these structures (Wilson et al., 1983; Gerfen, 1988), there has been a surge of interest in examining morphologic abnormalities of dendritic spines in various neurologic diseases (Penzes et al., 2011; Kuwajima et al., 2013). Most of our current knowledge about the alterations that occur in synaptic morphology in PD is derived from neurotoxin-induced rodent and primate models. Both 1-methyl-4-phenyl-1,2,3,6-tetrahydropyridine (MPTP) and 6-hydroxydopamine (6-OHDA), the commonly used neurotoxins for the generation of PD animal models, cause a marked decrease in the number of spines and an alteration in spine head volume in striatal dendrites (Solis et al., 2007; Villalba et al., 2009; Garcia et al., 2010; Naskar et al., 2013; Villalba and Smith, 2013, 2018; Funamizu et al., 2017; Gagnon et al., 2017). The structural abnormalities observed in neurotoxin-induced animal models are unlikely to be an artifact of drug application because postmortem analyses of PD patients’ brains have also revealed a severe loss of dendritic spines in this disease (McNeill et al., 1988; Stephens et al., 2005; Zaja-Milatovic et al., 2005).

Although neurotoxin-induced animal models nicely recapitulate the PD etiology observed in humans, the complete degeneration of dopaminergic cells in the substantia nigra pars compacta (SNc) after neurotoxin application suggests that these animal models mimic severe or late-stage PD. Similarly, postmortem analysis in humans usually represents the end stage of PD. Although informative, knowledge of structural changes in the final phase of PD are of limited use in the presymptomatic detection and early treatment of this disease. In addition, the knowledge obtained from neurotoxin-induced animal models may not necessarily represent a generalizable feature of PD. Importantly, the etiology of PD cannot be pinpointed to a single cause (Klein and Westenberger, 2012). Moreover, structural analysis of a genetically perturbed mouse model, the *lrrk2*-G2019S knock-in mouse, has revealed the presence of dendritic spines with a larger head volume than in the WT mouse but no change in spine density (Matikainen-Ankney et al., 2016). These results strongly indicate that structural changes observed in genetically perturbed animal models are distinct from those generated by pharmacological means.

Several studies have linked the α-synuclein gene, *SNCA*, to autosomal dominant PD, and at least six different point mutations (A30P, A53E, A53T, E46K, G51D, and H50Q) in *SNCA* have been identified in familial PD to date (Polymeropoulos et al., 1997; Krüger et al., 1998; Zarranz et al., 2004; Gasser, 2009; Lees et al., 2009; Appel-Cresswell et al., 2013; Kiely et al., 2013; Pasanen et al., 2014). A study has recently reported the generation of a novel A53T *SNCA* bacterial artificial chromosome (BAC) transgenic (Tg) mouse that demonstrates prodromal symptoms of PD such as hyposmia and rapid eye movement sleep behavior disorders, without any obvious motor dysfunction (Taguchi et al., 2020a). This mouse is therefore an excellent resource for studying structural changes in the early stages of PD. Here, we use focused ion beam/scanning electron microscopy (FIB/SEM) to study age-related structural changes in the A53T-BAC-*SNCA* Tg mouse model. Our results demonstrate that spine density decreases and spine head volume increases with age in wild-type (WT) mice, suggesting a homeostatic balance between spine density and spine head volume. However, in the A53T-BAC-*SNCA* Tg mouse, spine density was slightly higher than in the WT mouse of corresponding age, and the relative frequency of thin-type, presumably immature spines was also higher. Furthermore, the head volume of dendritic spines remained similar throughout the different developmental stages in A53T-BAC-*SNCA* Tg mice. Our results suggest that, in prodromal PD, distinct age-dependent synaptic structural changes occur.

## Materials and Methods

### Animals

Animal care and handling were performed in accordance with the animal welfare and experimental guidelines of the authors’ institutions. All experimental protocols were approved by the animal care and use committee of the authors’ institutions. The methodological details for the generation and genotyping of A53T-BAC-*SNCA* Tg mice are described in a previous study (Taguchi et al., 2020a). As in the previous study, heterozygous mice (hereafter referred to as A53T-BAC-*SNCA* mice) were used. For the present study, 16 male mice (eight mice for FIB/SEM analysis and eight mice for pre-embedding immunogold labeling) were used. For each experiment, one C57BL/6 WT mouse and one A53T-BAC-*SNCA* mouse were used at 1, 3, 6, and 22 months of age.

### Anesthesia, fixation, and sectioning

Mice were deeply anesthetized by inhalation of sevoflurane before being transcardially perfused with 20 ml of Ringer’s solution supplemented with 0.1% heparin. After the initial flushing out of blood from the vascular system, mice for FIB/SEM analysis were perfused with 100 ml of 2% paraformaldehyde (PFA) and 2% glutaraldehyde (GA) in 0.1 m cacodylate buffer. For pre-embedding immunogold labeling, ∼70 ml of fixative containing 4% PFA and 0.1% GA in 0.1 m phosphate buffer (PB; pH 7.4) was used. The perfusion speed was kept at 5–6 ml of fixative/min both for the FIB/SEM analysis and pre-embedding immunogold labeling. Each brain was extracted from the skull; for FIB/SEM analysis, brains were postfixed at 4°C overnight in the same fixative solution, while brains for pre-embedding immunogold labeling were subjected to 1 h of postfixation at RT in 4% PFA in 0.1 m PB. Following the thorough washing of brains in PBS, they were sectioned at 1 mm using a tissue chopper for FIB/SEM analysis and at 60 µm using a Linear Slicer Pro7 vibratome (Dosaka EM) for pre-embedding immunogold labeling.

### Tissue preparation for FIB/SEM

Brain blocks were rinsed several times in 0.1 m cacodylate buffer containing 2 mm CaCl_2_, and then incubated for 1 h in ferrocyanide-reduced osmium solution containing 1.5% potassium ferrocyanide and 2% aqueous osmium tetroxide (OsO_4_) in 0.1 m cacodylate buffer. Blocks were thoroughly rinsed in distilled water before being incubated for 2 h in 1% tannic acid solution. The sections were then incubated in 2% OsO_4_ for 30 min and in 1% aqueous uranyl acetate solution overnight. Following several washes with distilled water, brain blocks were dehydrated in 50%, 70%, 80%, 90%, and 100% ethanol for 10 min each. Blocks were then embedded in epoxy resin Epok812 (Okenshoji) and cured for 48 h at 60°C.

### FIB/SEM imaging

The striatal tissue surface was exposed using a Leica Ultracut UCT ultramicrotome. The block was then placed in a metal stub and sputter coated with gold-palladium to prevent specimen charging E1010 (E1010; Hitachi). Serial FIB/SEM images at 40–50 nm increments were acquired from the dorsolateral striatum on a Helios Nanolab 660 FIB/SEM using Auto Slice & View G3 software (FEI) to automate the serial milling and imaging process. The surface of the brain block was milled by the thermal energy produced by 0.77 nA of gallium (Ga) ion beam current that was accelerated at a voltage of 30 kV. The electron beam had a dwell time of 5 µs. The acceleration voltage in the backscattered electron detector was set to 2.0 kV with 0.8 nA. Images were obtained at 15,000× magnification covering a distance of 13.82 µm in the horizontal direction and 11.69 µm in vertical direction at a resolution of 4.5 nm/pixel. The lateral resolution (*x*, *y*) and axial resolution (*z*, section thickness) used in our study is within the typical range used for electron microscopic (EM) imaging of neuronal structures. At this resolution, we can clearly resolve synaptic vesicles, postsynaptic density (PSD), small spines and thin axons.

### Three-dimensional (3D) reconstruction and morphologic quantification of spines and dendrites

3D reconstruction and analysis of spine and dendrite morphology was performed in accordance with Sato and Okabe (2019). Briefly, FIB/SEM images were first automatically aligned using Fiji software (Schindelin et al., 2012). Image stacks were then loaded onto Reconstruct software (Fiala, 2005) and dendritic shafts and spines were manually segmented. The numerical values for the surface area and the volume of dendritic spines were obtained from the Reconstruct software. PSD area was obtained by multiplying the section thickness with the summed length of PSD in consecutive sections. Spine neck length and dendritic length were obtained in 3D-reconstructed images in reference to a square of 1 µm in length on each side. Spine density was obtained by dividing the total number of spines in a dendrite by the dendritic length.

### Pre-embedding immunogold labeling

Sections from the dorsolateral striatum of WT and A53T-BAC-*SNCA* mice were rinsed briefly in 25 mm PBS (25 mm PB and 150 mm NaCl) and then incubated in a cryoprotectant solution containing 25% sucrose and 10% glycerol in 0.1 m PB. Sections were then permeabilized by repeated cycles of freezing in liquid nitrogen and thawing. Thereafter, the sections were washed three times for 20 min in 50 mm Tris-buffered saline (TBS; 50 mm Tris-HCl, pH 7.4, and 150 mm NaCl) and incubated in a blocking solution containing 10% normal goat serum (NGS) in TBS for 1 h. The sections were then incubated for 48 h in rabbit monoclonal anti-phosphorylated α-synuclein antibody (phospho-S129, clone EP1536Y, catalog number ab51253; Abcam) at a dilution of 1:1000 in 2% NGS in TBS. The sensitivity and specificity of this antibody was confirmed in a previous study (Delic et al., 2018). Sections were then washed three times for 20 min each with TBS and incubated overnight at 4°C in 1.4 nm nanogold-conjugated goat anti-rabbit secondary antibody (Nanoprobes). The sections were washed in TBS and fixed with 1% GA in PBS for 10 min. Following several washes in PBS, sections were briefly rinsed in distilled water, and immunogold signals were then enhanced using an HQ silver enhancement kit (Nanoprobes). The sections were postfixed in 1% OsO_4_ for 40 min, incubated with uranyl acetate for 35 min, dehydrated in a graded series of ethanol, subjected to two changes of propylene oxide, and then immersed in Durcupan resin (ACM Fluka, Sigma-Aldrich) overnight. Sections were then flat-embedded between glass slides and cured for 48 h at 60°C. Ultrathin sections (70 nm) were cut with a Leica Ultracut UCT ultramicrotome. Ultrathin sections were stained for 1 min with 1% lead citrate and observed with a Hitachi HT7700 transmission electron microscope (TEM).

### Statistics

Statistical analysis was performed using IBM SPSS statistics software (IBM, version 24). The normality of the datasets was assessed using the Shapiro–Wilk test. Statistical differences between two datasets were examined using a Student’s *t* test or Mann–Whitney *U* test, depending on the outcome of the normality analysis. To examine statistical differences between three or more groups, we used one-way ANOVA for parametric datasets and the Kruskal–Wallis test for non-parametric datasets. The distribution of spine head volume among various ages was analyzed by Kolmogorov–Smirnov *Z* test. Statistical correlation between two datasets was examined using the Spearman’s rank order test. Unless otherwise mentioned, data are expressed as mean ± SEM.

## Results

### Reconstruction of striatal dendrites and spines from FIB/SEM

**Figure 1.**
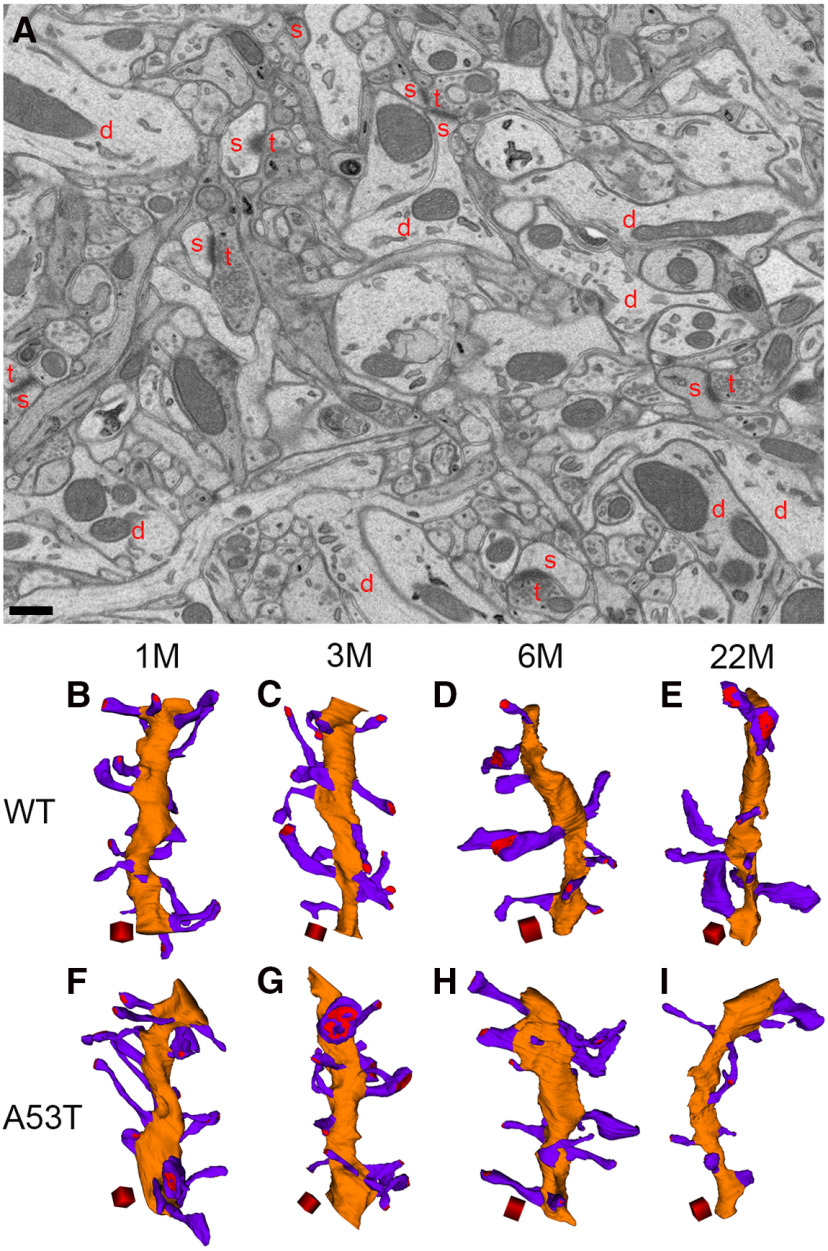
3D reconstruction of dendrites and spines from WT and A53T-BAC-*SNCA* mice at different ages. ***A***, Membrane contours of dendrites (d), spines (s), and presynaptic terminals (t) can be clearly observed in the FIB/SEM image from a 1-month-old WT mouse. Mitochondria and other organelles are also clearly visible. Scale bar: 500 nm. ***B–I***, 3D reconstruction of dendrites (orange) and spines (violet) from WT mice at 1 (***B***), 3 (***C***), 6 (***D***), and 22 (***E***) months of age and from *A53T-BAC-SNCA* mice at 1 (***F***), 3 (***G***), 6 (***H***), and 22 (***I***) months of age. Red regions in the spine heads indicate postsynaptic densities. Scale cubes: 0.5 µm on each side.

Our sample preparation and imaging protocol produced FIB/SEM images with high structural integrity, and the membrane contours of dendrites, spines, and presynaptic axons were clearly visible. Synaptic contacts and PSDs of excitatory synapses were also clearly discernible (Fig. 1*A*). Using ∼250–400 serial FIB/SEM images (number of sections imaged for WT at 1 month = 374, 3 months = 360, 6 months = 333, 22 months = 396; number of sections imaged for A53T-BAC-*SNCA* at 1 month = 253, 3 months = 295, 6 months = 382, 22 months = 380), we could reconstruct approximately a 5- to 25-µm length of dendrites, including all the spines protruding from that dendritic segment. We analyzed 65 dendrites and 1285 spines in this study. We reconstructed seven dendrites and 132 spines from a 1-month-old WT mouse, seven dendrites and 148 spines from a 1-month-old A53T-BAC-*SNCA* mouse, eight dendrites and 177 spines from a 3-month-old WT mouse, eight dendrites and 251 spines from a 3-month-old A53T-BAC-*SNCA* mouse, seven dendrites and 109 spines from a 6-month-old WT mouse, seven dendrites and 151 spines from a 6-month-old A53T-BAC-*SNCA* mouse, 11 dendrites and 156 spines from a 22-month-old WT mouse, and 10 dendrites and 161 spines from a 22-month-old A53T-BAC-*SNCA* mouse. The average length (in µm) of reconstructed dendrites was 9.2 ± 0.61 (median = 8.6, range = 8.0–12.5) in a 1-month-old WT mouse, 8.7 ± 0.75 (median = 8.7, range = 5.4–10.9) in a 1-month-old A53T-BAC-*SNCA* mouse, 12.4 ± 0.52 (median = 13.2, range = 9.8–13.9) in a 3-month-old WT mouse, 13.8 ± 1.20 (median = 13.3, range = 9.7–19.5) in a 3-month-old A53T-BAC-*SNCA* mouse, 11.8 ± 0.85 (median = 10.5, range = 9.4–14.5) in a 6-month-old WT mouse, 11.0 ± 0.72 (median = 11.4, range = 7.8–12.9) in a 6-month-old A53T-BAC-*SNCA* mouse, 13.4 ± 0.83 (median = 13.7, range = 8.6–17.6) in a 22-month-old WT mouse, and 11.7 ± 1.49 (median = 11.0, range = 7.2–24.1) in a 22-month-old A53T-BAC-*SNCA* mouse.

Each dendrite contained spines with varying dimensions of head volume and neck length. Figure 1*B-I* shows representative dendrites reconstructed from each mouse. Qualitative inspection of the 3D-reconstructed dendritic segments gave the impression that, in WT mice, spine abundance in dendrites decreases with age, while head volume of spines increases with age (Fig. 1*B–E*). Numerous small spines were predominant in the 1-month-old mouse, while large spines outnumbered small spines in the 6- and 22-month-old WT mice. Similar to the trend observed in WT mice, an age-related decline in dendritic spine abundance was also observed in the mutant mice. However, an increase in spine head volume with age was not obvious in A53T-BAC-*SNCA* mice (Fig. 1*F–I*).

### Alterations in dendritic spine ultrastructure in A53T-BAC-*SNCA* mice

**Figure 2.**
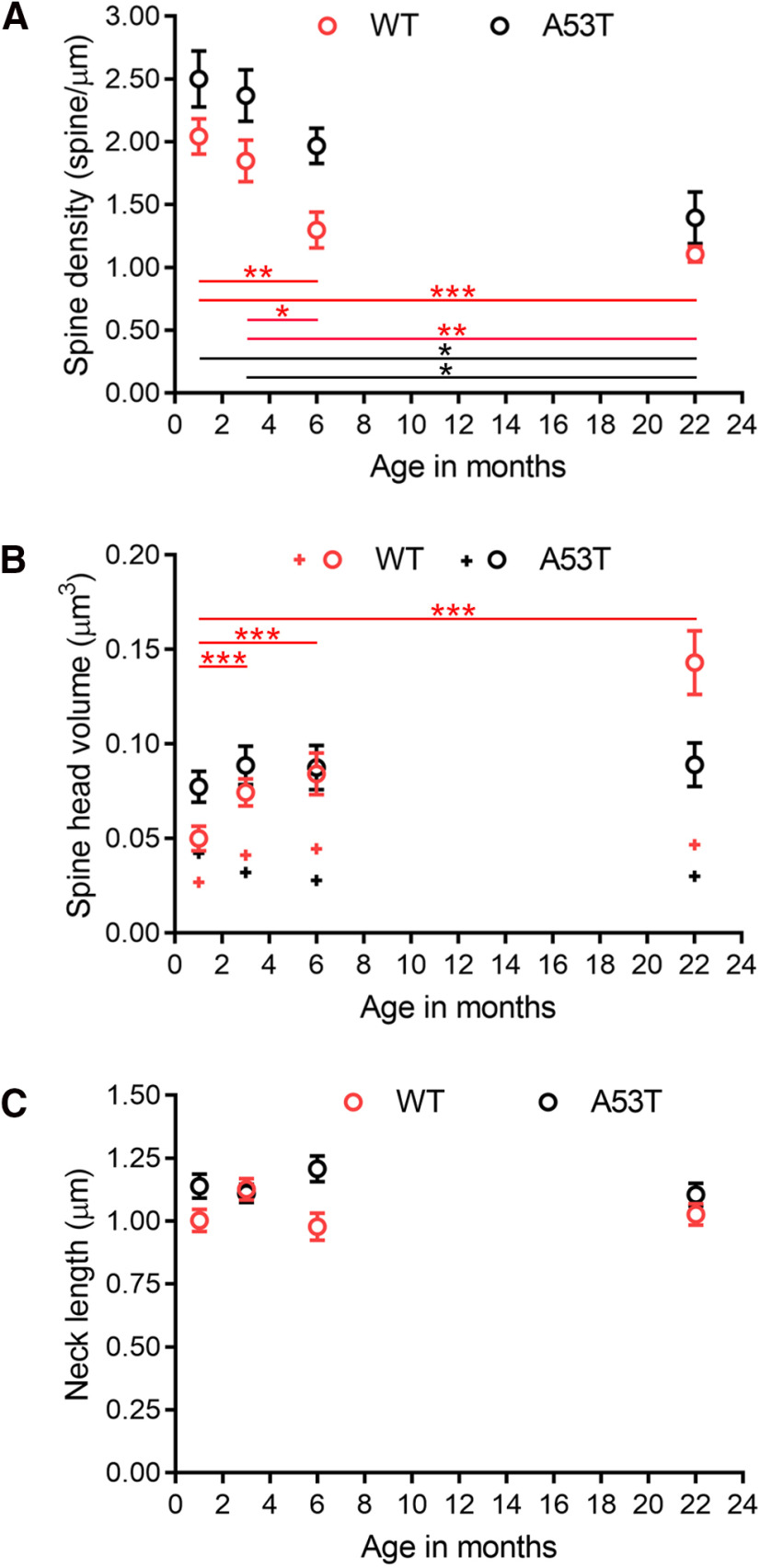
Morphologic alterations in spines in WT and A53T-BAC-*SNCA* mice with age. ***A***, Age-related changes in spine density. The graph shows that spine density decreased with age in both the WT and A53T-BAC-*SNCA* mice. In WT mice, the spine density at 1 and 3 months was significantly lower compared with that at 6 and 22 months (**p =* *0.003 between 1 and 6 months, ***p <* *0.001 between 1 and 22 months, *p =* *0.03 between 3 and 6 months, **p =* *0.001 between 3 and 22 months; one-way ANOVA). In A53T-BAC-*SNCA* mice, there were fewer spines at 22 months compared with that at 1 and 3 months (*p =* *0.01 between 1 and 22 months, *p =* *0.01 between 3 and 22 months; Kruskal–Wallis test). ***B***, Age-related changes in spine head volume. The average spine head volume increases with age in the WT (red open circle), but not A53T-BAC-*SNCA* (black open circle), mice. In WT mice, spine head volume at 1 month was significantly smaller than that at 3, 6, and 22 months (***p <* *0.001 between 1 and 3 months, ***p < 0.001 between 1 and 6 months, ***p <* *0.001 between 1 and 22 months; Kruskal–Wallis test). Median head volume is shown by the + symbol. ***C***, Age-related changes in spine neck length. The spine neck length did not vary significantly with age in either WT or A53T-BAC-*SNCA* mice. Different levels of significance are denoted by the number of asterisks (*p <0.05, **p <0.01, ***p <0.001).

To precisely understand the structural abnormalities in A53T-BAC-*SNCA* mice, we performed an extensive analysis of serial FIB/SEM images to obtain quantitative data for various parameters that can influence synaptic changes. In a dendrite, the density of spines, the volume of spine heads, and the length of spine necks all critically influence the strength of synaptic transmission (Tønnesen and Nägerl, 2016; Parajuli et al., 2017). Thus, we quantified and compared these parameters for each age and genotype. In WT mice, the mean spine densities (expressed as the number of spines/µm length of dendrite) in dendrites were 2.0 ± 0.14 (median = 1.9, range = 1.6–2.6, *n *=* *132 spines, seven dendrites), 1.8 ± 0.17 (median = 1.9, range = 1.4–2.9, *n *=* *177 spines, eight dendrites), 1.3 ± 0.14 (median = 1.3, range = 0.7–2.0, *n *=* *109 spines, seven dendrites), and 1.1 ± 0.06 (median = 1.0, range = 0.8–1.4, *n *=* *156 spines, 11 dendrites) at 1, 3, 6, and 22 months of age, respectively (Fig. 2*A*). Spine densities at 6 and 22 months of age were significantly lower than at 1 and 3 months of age (*p* values between 1 and 6 months, *p* = 0.003; 1 and 22 months, *p* < 0.001; 3 and 6 months, *p* = 0.03; 3 and 22 months, *p* = 0.001; one-way ANOVA). In contrast, there was no significant difference in spine density between 1 and 3 months of age (*p *=* *1.00; one-way ANOVA) or 6- and 22-month-old mice (*p *=* *1.00; one-way ANOVA). Spine densities in A53T-BAC-*SNCA* mice also trended downward with age (Fig. 2*A*). Mean dendritic spine densities were 2.5 ± 0.22 (median = 2.4, range = 1.7–3.5, *n *=* *148 spines, seven dendrites), 2.4 ± 0.21 (median = 2.5, range = 1.3–3.0, *n *=* *251 spines, eight dendrites), 2.0 ± 0.14 (median = 2.0, range = 1.5–2.5, *n *=* *151 spines, seven dendrites), and 1.4 ± 0.21 (median = 1.2, range = 0.6–3.1, *n *=* *161 spines, 10 dendrites) at 1, 3, 6, and 22 months of age, respectively. In the A53T-BAC-*SNCA* mouse, spine densities in striatal dendrites at 1 and 3 months of age were significantly lower than at 22 months of age (*p* values between 1 and 22 months, *p* = 0.01; 3 and 22 months, *p* = 0.01; Kruskal–Wallis test). There were no significant differences in spine density in the A53T-BAC-*SNCA* mice between 1 and 3 months of age (*p *=* *1.00; Kruskal–Wallis test), 1 and 6 months of age (*p *=* *1.00; Kruskal–Wallis test), 3 and 6 months of age (*p *=* *1.00; Kruskal–Wallis test) and 6 and 22 months of age (*p *=* *0.48; Kruskal–Wallis test). A comparison between WT and A53T-BAC-*SNCA* mice of corresponding ages revealed a significant difference in spine density at 6 months of age (*p* value between WT and A53T-BAC-*SNCA* mice, *p* = 0.006; Student’s *t* test). However, there were no significant differences between mice at 1 month of age (*p* value between WT and A53T-BAC-*SNCA* mice, *p* = 0.11; Student’s *t* test), at 3 months of age (*p* value between WT and A53T-BAC-*SNCA* mice, *p* = 0.07; Student’s *t* test), and at 22 months of age (*p* value between WT and A53T-BAC-*SNCA* mice = 0.17; Mann–Whitney *U* test). These data suggest that, in the A53T-BAC-*SNCA* mouse, the mechanisms that regulate either spinogenesis or the maintenance of dendritic spines at 6 months of age are impaired.

In contrast to the trend of decreasing spine density with age, there was an increase in average spine head volume with age in WT mice (Fig. 2*B*). The average spine head volumes (in µm^3^) in WT mice were 0.050 ± 0.0065 (median = 0.027, range = 0.001–0.539, *n *=* *132 spines), 0.074 ± 0.0071 (median = 0.041, range = 0.006–0.688, *n *=* *177 spines), 0.084 ± 0.0110 (median = 0.045, range = 0.006–0.778, *n *=* *109 spines), and 0.143 ± 0.0169 (median = 0.047, range = 0.005–1.151, *n *=* *156 spines) at 1, 3, 6, and 22 months of age, respectively. The spine head volumes were significantly larger at 3, 6, and 22 months of age than at 1 month of age (*p* values between 1 and 3 months, *p* < 0.001; 1 and 6 months, *p* < 0.001; 1 and 22 months, *p* < 0.001; Kruskal–Wallis test). The median head volume of dendritic spines in the striatum remained similar among 3, 6, and 22 months of age, and there was no statistically significant difference in spine head volume among these three ages (*p* values between 3 and 6 months, *p* = 1.00; 3 and 22 months, *p* = 1.00; 6 and 22 months, *p* = 1.00; Kruskal–Wallis test). In contrast to what was observed in the WT mice, the average spine head volume in the A53T mutant remained relatively similar across the different ages. The average spine head volumes (in µm^3^) in the A53T-BAC-*SNCA* mice were 0.077 ± 0.0082 (median = 0.042, range = 0.005–0.547, *n *=* *148 spines), 0.089 ± 0.0102 (median = 0.032, range = 0.005–1.286, *n *=* *251 spines), 0.088 ± 0.0116 (median = 0.028, range = 0.005–0.782, *n *=* *151 spines), and 0.089 ± 0.0115 (median = 0.030, range = 0.003–1.138, *n *=* *161 spines) at 1, 3, 6, and 22 months of age, respectively. Spine head volumes were not significantly different among these four different ages (*p* values between 1 and 3 months, *p* = 0.07; 1 and 6 months, *p* = 0.06; 1 and 22 months, *p* = 0.11; 3 and 6 months, *p* = 1.00; 3 and 22 months, *p* = 1.00; 6 and 22 months, *p* = 1.00; Kruskal–Wallis test). A comparison between WT and A53T-BAC-*SNCA* mice of corresponding ages revealed that there was a significant difference in spine head volume at each age (*p* values between WT and A53T-BAC-*SNCA* at 1 month, *p* < 0.001; at 3 months, *p* = 0.008; at 6 months, *p* = 0.04; at 22 months, *p* = 0.006; Mann–Whitney *U* test). These data suggest that factors that regulate spine head volume is impaired throughout different developmental stages in A53T-BAC-*SNCA* mice.

Spine neck is also an important parameter governing the magnitude of synaptic transmission across synaptic contacts. In particular, the dimension of spine neck length governs the degree of attenuation in membrane potential when it travels from the spine head toward its parent dendrite (Araya et al., 2006). Thus, we obtained the average, median, and range of spine neck lengths across different ages both in the WT and A53T-BAC-*SNCA* mice. The average spine neck length (in µm) in WT mice were 1.00 ± 0.045 (median = 0.96, range = 0.09–2.30, *n *=* *132 spines), 1.13 ± 0.043 (median = 1.10, range = 0.09–3.18, *n *=* *176 spines), 0.98 ± 0.054 (median = 0.92, range = 0.09–2.71, *n *=* *108 spines), and 1.03 ± 0.043 (median = 0.95, range = 0.08–2.90, *n *=* *156 spines) at 1, 3, 6, and 22 months of age, respectively (Fig. 2*C*). Spine neck lengths were not significantly different across developmental stages in WT mice (*p* values between 1 and 3 months, *p* = 0.48; 1 and 6 months, *p* = 1.00; 1 and 22 months, *p* = 1.00; 3 and 6 months, *p* = 0.07; 3 and 22 months, *p* = 0.46; and 6 and 22 months, *p* = 1.00; Kruskal–Wallis test). The average spine neck length in A53T-BAC-*SNCA* mice was also relatively similar across different ages. The average spine neck lengths (in µm) in A53T-BAC-*SNCA* mice were 1.14 ± 0.047 (median = 1.07, range = 0.07–3.16, *n *=* *148 spines), 1.11 ± 0.036 (median = 1.03, range = 0.17–3.30, *n *=* *249 spines), 1.21 ± 0.052 (median = 1.14, range = 0.05–3.10, *n *=* *149 spines), and 1.11 ± 0.045 (median = 1.09, range = 0.10–2.64, *n *=* *159 spines) at 1, 3, 6, and 22 months of age, respectively. Spine neck lengths were not significantly different among these four different ages (*p* values between 1 and 3 months, *p* = 1.00; 1 and 6 months, *p* = 1.00; 1 and 22 months, *p* = 1.00; 3 and 6 months, *p* = 1.00; 3 and 22 months, *p* = 1.00; and 6 and 22 months, *p* = 1.00; Kruskal–Wallis test). When the spine neck length was compared between WT and A53T-BAC-*SNCA* mice of corresponding ages, there was a significant difference at 6 months of age only (*p* values between WT and A53T-BAC-*SNCA* mice at 1 month, *p* = 0.06; at 3 months, *p* = 0.71; at 6 months, *p* = 0.002; at 22 months, *p* = 0.15; Mann–Whitney *U* test). Together, these results demonstrate that each developmental stage is accompanied by distinct changes in spine morphology. Although spine head volume was altered in A53T-BAC-*SNCA* mice at all examined ages, spine neck length and spine density were only higher in A53T-BAC-*SNCA* mice at 6 months of age.

### Presence of large dendritic spines in aged WT but not A53T-BAC-*SNCA* mice

Despite the large apparent difference in mean spine head volume in WT mice between 6 and 22 months of age, there was no statistically significant difference between these two ages because of similarities in the median spine head volume. Theoretically, although median values are similar, mean spine head volume may be much larger in one group if it contains a number of particularly large spines. To explore this issue, we therefore assessed the relative frequency of small and large spines by broadly categorizing spines into two groups based on their spine head volume. A cutoff volume of 0.04 µm^3^ was chosen to group the spines into either large or small types. As the median head volume in adult WT mice (3 and 6 months of age) was ∼0.04 µm^3^, we decided to choose this value as the cutoff to group spines into small and large types. This criterion will split spines into approximately two equal halves with 50% of the spines whose head volume is below the median head volume as small spines and 50% of the spines whose head volume is above the median head volume as large spines. Based on these criteria, we observed that in WT mice, relative frequency of small spines was 72.7% at 1 month of age (*n *=* *96 out of 132), 48.6% at 3 months of age (*n *=* *86 out of 177), 45.0% at 6 months of age (*n *=* *49 out of 109), and 44.2% at 22 months of age (*n *=* *69 out of 156). The relative frequency of small spines at 1 month was higher than that at 3, 6, and 22 months of age. Conversely, the relative frequencies of large spines at 3, 6, and 22 months of age were higher than at 1 month. Relative frequency of large spines was 27.3% at 1 month of age (*n *=* *36 out of 132), 51.4% at 3 months of age (*n *=* *91 out of 177), 55.0% at 6 months of age (*n *=* *60 out of 109), and 55.8% at 22 months of age (*n *=* *87 out of 156). The relative frequencies of small and large spines in WT mice remained fairly similar among 3, 6, and 22 months of age, suggesting that the relative abundance of large and small spines in WT mice considerably changes between 1 and 3 months of age.

The relative frequencies of small and large spines in A53T-BAC-*SNCA* mice were different from those in WT mice. In A53T-BAC-*SNCA* mice, higher frequencies of small spines (and a corresponding lower frequency of large spines) were observed at 3, 6, and 22 months of age than at 1 month. At 1, 3, 6, and 22 months of age, respectively, small spines made up 47.3% (70 out of 148), 57.8% (145 out of 251), 60.9% (92 out of 151), and 57.8% (93 out of 161) of total spines. Our data therefore show a higher frequency of mature spines in WT mice than in A53T-BAC-*SNCA* mice at 3, 6, and 22 months of age.

When all the spine population was taken into consideration, the spine head volume in WT mice at 3, 6, and 22 months were not statistically significant (Fig. 2*B*). Next, we wanted to examine if the head volume of either small or large spines differs with age. The mean head volume (in µm^3^) of small spines was 0.025 ± 0.0010 (median = 0.026, range = 0.006–0.040, *n *=* *86 spines), 0.021 ± 0.0013 (median = 0.023, range = 0.006–0.040, *n *=* *49 spines), and 0.020 ± 0.0013 (median = 0.019, range = 0.005–0.040, *n *=* *69 spines) at 3, 6, and 22 months of age, respectively. The spine head volume of small spines was significantly larger at 3 months of age than at 22 months of age (*p* value between 3 and 22 months, *p* = 0.009; Kruskal–Wallis test). No statistical significance was detected in the spine head volume of small spines between 3 and 6 months or 6 and 22 months (*p* values between 3 and 6 months, *p* = 0.10; 6 and 22 months, *p* = 1.00; Kruskal–Wallis test). The mean head volume (in µm^3^) of large spines was 0.121 ± 0.0120 (median = 0.073, range = 0.041–0.688, *n *=* *91 spines), 0.135 ± 0.0175 (median = 0.094, range = 0.040–0.778, *n *=* *60 spines), and 0.241 ± 0.0259 (median = 0.130, range = 0.040–1.151, *n *= 87 spines) at 3, 6, and 22 months of age, respectively. The spine head volume of large spines was significantly larger at 22 months of age than at 3 and 6 months of age (*p* values between 3 and 22 months, *p* = 0.001; 6 and 22 months, *p* = 0.04; Kruskal–Wallis test). In contrast, neither the head volume of small spines nor the large spines showed statistical significance among 3, 6, and 22 months of ages in A53T-BAC-*SNCA* mice. The mean head volume (in µm^3^) of small spines in A53T-BAC-*SNCA* was 0.020 ± 0.0007 (median = 0.018, range = 0.005–0.040, *n *= 145 spines), 0.020 ± 0.0009 (median = 0.019, range = 0.005–0.038, *n *=* *92 spines), and 0.019 ± 0.0010 (median = 0.019, range = 0.003–0.039, *n *=* *93 spines) at 3, 6, and 22 months of age, respectively (*p* values between 3 and 6 months, *p* = 1.00; 3 and 22 months, *p* = 0.82; 6 and 22 months, *p* = 1.00; Kruskal–Wallis test). The mean head volume (in µm^3^) of large spines in A53T-BAC-*SNCA* was 0.182 ± 0.0211 (median = 0.094, range = 0.040–1.286, *n *= 106 spines), 0.194 ± 0.0239 (median = 0.112, range = 0.041–0.782, *n *=* *59 spines), and 0.185 ± 0.0225 (median = 0.094, range = 0.040–1.138, *n *=* *68 spines) at 3, 6, and 22 months of age, respectively (*p* values between 3 and 6 months, *p* = 0.47; 3 and 22 months, *p* = 0.70; 6 and 22 months, *p* = 1.00; Kruskal–Wallis test).

**Figure 3.**
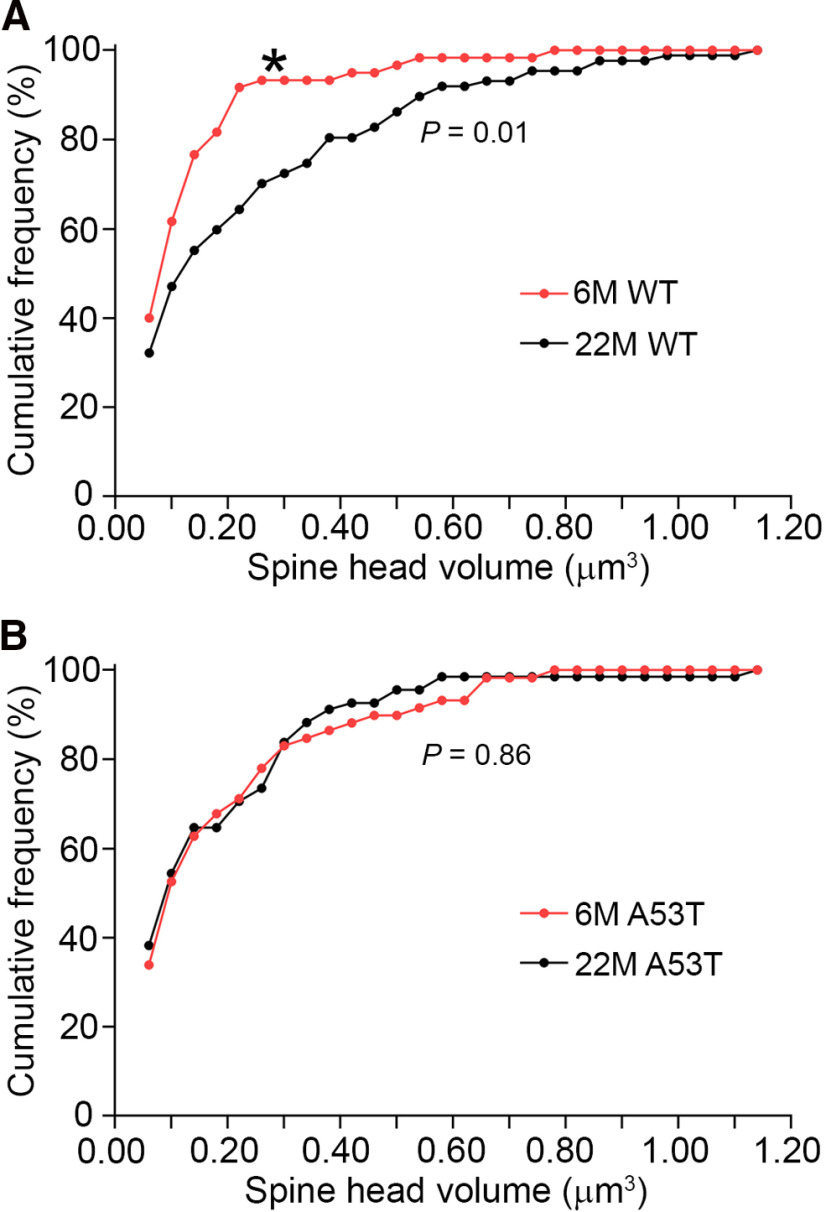
The frequency of large spines increased with age in WT, but not A53T-BAC-*SNCA*, mice. ***A***, The distribution of large spines in WT mice was significantly different between 6 and 22 months of age (*p =* *0.01, Kolmogorov–Smirnov test). ***B***, In contrast, the distribution of large spines was not significantly different between 6 and 22 months of age in A53T-BAC-*SNCA* mice (*p *=* *0.86, Kolmogorov–Smirnov test).

In order to compare the distribution of spine head volume of mushroom-type spines between 6 and 22 months of ages in WT mice, we binned the head volume of mushroom-type spines into a bin-width of 0.04 µm^3^ and plotted the relative frequency of spines in each bin (Fig. 3*A*). The distribution of spine head volume in the 22-month-old mouse showed a rightward shift in comparison to that of the 6-month-old mouse (*p *=* *0.01, Kolmogorov–Smirnov *Z* test). In contrast, spine head volume of the large spines was not statistically different between the 6- and 22-month-old A53T-BAC-*SNCA* mice (*p *=* *0.86, Kolmogorov–Smirnov *Z* test; Fig. 3*B*). These data further reiterate that aging is accompanied by an increase in the frequency of large spines in WT mice.

We also compared the neck length between large and small spines at 3, 6, and 22 months of age. The neck length (in µm) of small spines in WT was 1.31 ± 0.061 (median = 1.26, range = 0.20–3.14, *n *=* *85 spines), 1.00 ± 0.082 (median = 0.97, range = 0.09–2.71, *n *=* *48 spines), 1.20 ± 0.072 (median = 1.09, range = 0.16–2.90, *n *=* *69 spines) at 3, 6, and 22 months of age, respectively. The neck length (in µm) of large spines in WT was 0.95 ± 0.054 (median = 0.91, range = 0.09–3.18, *n *=* *91 spines), 0.96 ± 0.073 (median = 0.87, range = 0.18–2.69, *n *=* *60 spines), and 0.89 ± 0.048 (median = 0.77, range = 0.08–1.89, *n *=* *87 spines) at 3, 6, and 22 months of age, respectively. The neck length of large spines was significantly shorter than that of small spines at 3 and 22 months of age in WT (*p* values between neck lengths of small and large spines at 3 months, *p* < 0.001; 6 months, *p* = 0.61; 22 months, *p* = 0.001; Mann–Whitney *U* test). The neck length (in µm) of small spines in A53T-BAC-*SNCA* was 1.14 ± 0.048 (median = 1.08, range = 0.18–2.58, *n *=* *143 spines), 1.26 ± 0.069 (median = 1.21, range = 0.05–3.10, *n *=* *90 spines), and 1.12 ± 0.063 (median = 1.09, range = 0.10–2.64, *n *=* *92 spines) at 3, 6, and 22 months of age, respectively. The neck length (in µm) of large spines in A53T-BAC-*SNCA* was 1.06 ± 0.055 (median = 1.01, range = 0.17–3.30, *n *=* *106 spines), 1.13 ± 0.076 (median = 1.05, range = 0.26–2.48, *n *=* *59 spines), and 1.08 ± 0.063 (median = 1.12, range = 0.14 –2.22, *n *=* *67 spines) at 3, 6, and 22 months of age, respectively. In contrast to that observed in WT, no statistical significance was detected in the neck lengths between small and large spines at any of the ages in A53T-BAC-*SNCA* (*p* values between neck lengths of small and large spines at 3 months, *p* = 0.22; 6 months, *p* = 0.19; 22 months, *p* = 0.81; Mann–Whitney *U* test). Our data show that A53T-BAC-*SNCA* mice show deficits in normal spine developmental processes and maturation.

### Higher frequency of perforated spines in WT mice at 22 months

**Figure 4.**
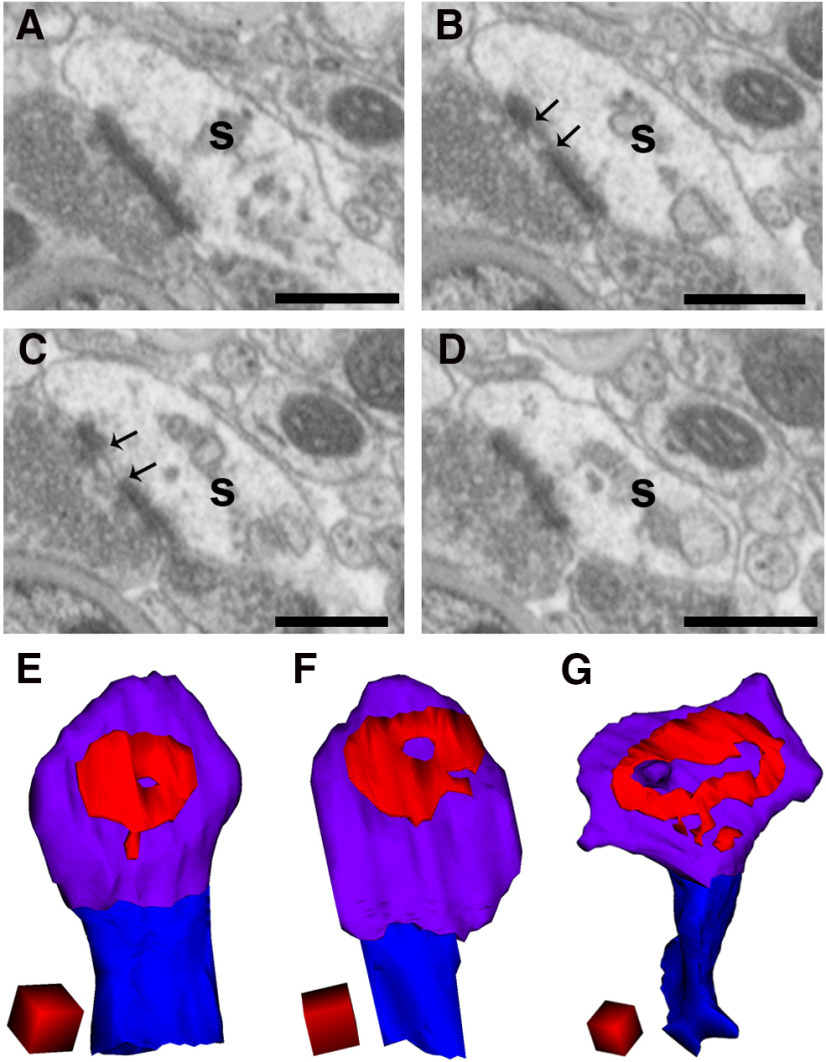
Examples of perforated spines in WT mice at 22 months of age. ***A–D***, Serial images of a large spine (s). Arrows in ***B***, ***C*** show the discontinuity of the PSD. Scale bars: 500 nm. ***E–G***, Representative images of a 3D reconstruction of spines showing a doughnut-shaped perforated PSD with a hole at the center. Spine heads, spine necks, and PSDs are shown in violet, blue, and red, respectively. Scale cubes: 0.25 µm on each side.

The PSDs of spines are broadly classified into macular and perforated types based on either the absence or presence of discontinuity in the PSD. While PSDs in thin-type spines are known to be predominantly macular, a subset of large or mushroom-type spines possess perforated PSDs (Geinisman et al., 1986). In our serial FIB/SEM images, we occasionally encountered spines with PSDs that showed discontinuity (Fig. 4*A–D*). In the 3D reconstructions, some of the perforated PSDs were doughnut-shaped with a hole in the center (Fig. 4*E–G*). Quantification revealed that the percentages of perforated spines in the WT mice were 1.52%, 4.52%, 6.42%, and 21.15% at 1, 3, 6, and 22 months, respectively. The percentage of perforated spines at 22 months was 3.29-fold higher than at 6 months, 4.68-fold higher than at 3 months, and 13.91-fold higher than at 1 month. In the A53T-BAC-*SNCA* mice, the percentages of perforated spines were 2.70%, 9.96%, 5.96%, and 9.94% at 1, 3, 6, and 22 months, respectively. Furthermore, the frequency of perforated spines at 22 months of age was just 1.67-fold higher than at 6 months and 3.68-fold higher than at 1 month in the A53T-BAC-*SNCA* mice. There was hardly any difference in the frequency of perforated spines between 3 and 22 months of age in A53T-BAC-*SNCA* mice. In addition, the percentage of perforated spines was approximately 2-fold larger in WT mice than in A53T-BAC-*SNCA* mice at 22 months of age. This suggests that spine perforation increases with age in WT mice, and that an abnormality exists in either the formation, maintenance, or elimination of perforated spines in A53T-BAC-*SNCA* mice.

### The linear relationship between spine head volume and PSD area is maintained in A53T-BAC-*SNCA* mice

**Figure 5.**
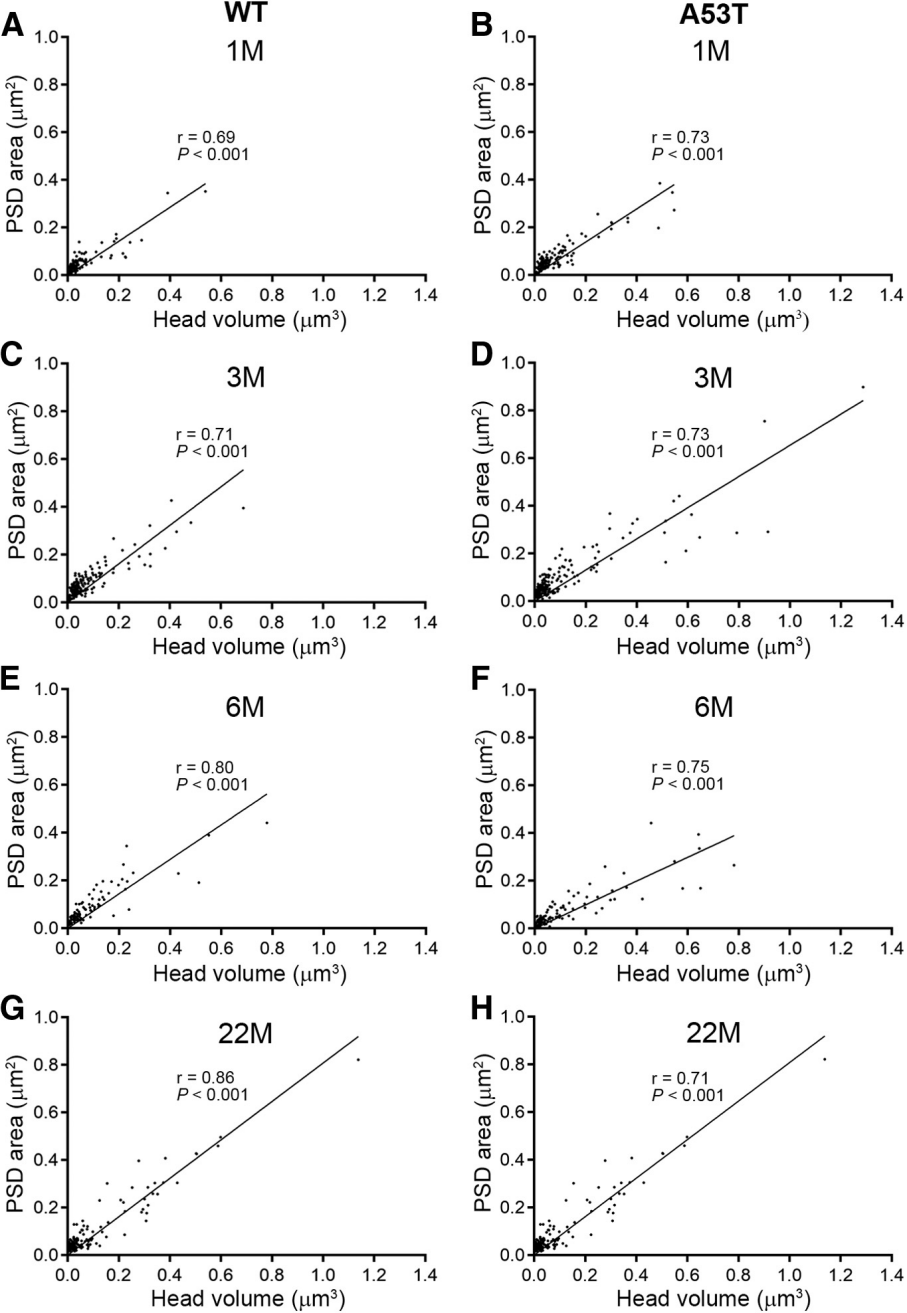
PSD area was correlated with spine head volume in both WT and A53T-BAC-*SNCA* mice. ***A–H***, A significant, positive linear correlation exists between PSDs and spine head volumes in both WT (***A***, ***C***, ***E***, ***G***) and A53T-BAC-*SNCA* (***B***, ***D***, ***F***, ***H***) mice at all four examined ages [1 month (***A***, ***B***); 3 months (***C***, ***D***); 6 months (***E***, ***F***); 22 months (***G***, ***H***)]. The correlation coefficients and the *p* values for the test of statistical significance of correlation are denoted by *r* and *p*, respectively, in the corresponding graphs. Correlation was examined using Spearman’s rank order test.

It has been well-established that spine head volume is positively correlated with both PSD area and AMPA receptor content (Wilson et al., 1983; Matsuzaki et al., 2001). Thus, spine head volume is often considered a proxy for synaptic strength. Because the A53T-BAC-*SNCA* mouse demonstrated abnormal development and maturation of spine head volume, we investigated whether the linear relationship between head volume and PSD area was disrupted in A53T-BAC-*SNCA* mice. To do this, we plotted the head volume of spines against their PSD area. The plot revealed a strong and significant correlation between head volume and PSD area, both in WT (Fig. 5*A*,*C*,*E*,*G*) and A53T-BAC-*SNCA* (Fig. 5*B*,*D*,*F*,*H*) mice at all four ages examined. Furthermore, the slope of the regression line was not considerably different between the WT and A53T-BAC-*SNCA* mice. Taken together, these data demonstrate that the basic architectural relationship between spine head volume and PSD area is not altered in A53T-BAC-*SNCA* mice.

### Aggregation of phosphorylated α-synuclein in a subset of presynaptic terminals in A53T-BAC-*SNCA* mice

**Figure 6.**
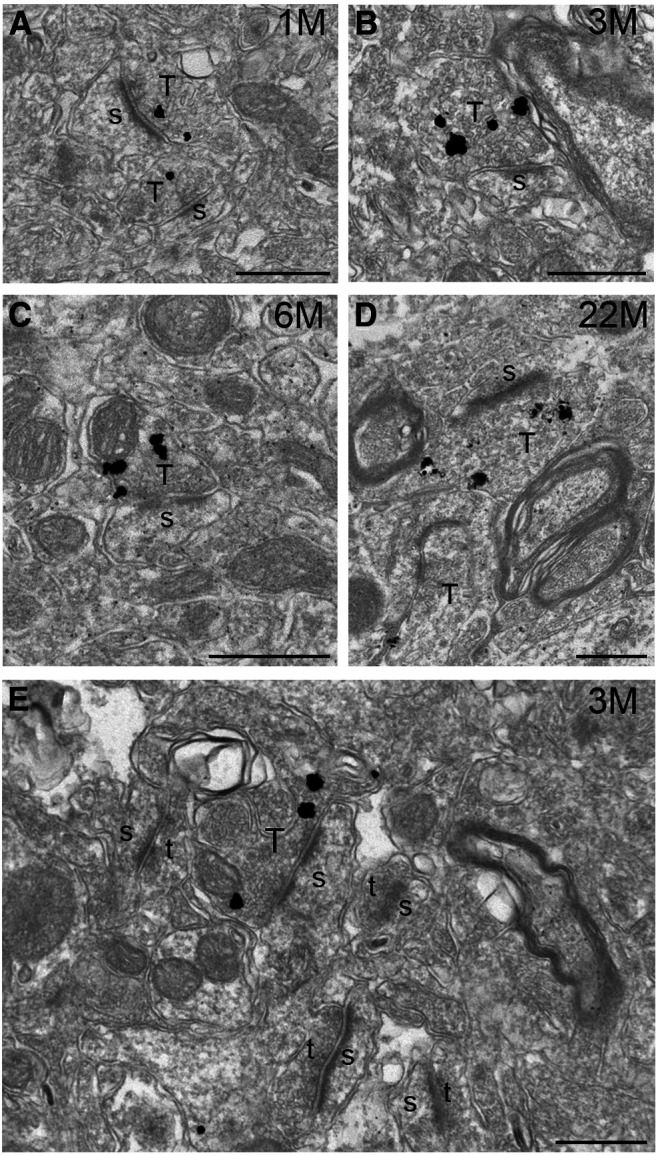
Localization of phosphorylated α-synuclein in a subset of presynaptic terminals in A53T-BAC-*SNCA* mice. ***A–D***, TEM images of pre-embedding immunogold-labeled samples from A53T-BAC-*SNCA* mice at 1 (***A***), 3 (***B***), 6 (***C***), and 22 (***D***) months. Note that the immunogold particles, indicating phosphorylated α-synuclein, were predominantly localized to presynaptic terminals. Immunogold particles were often seen in clusters. ***E***, A low-magnification image from an A53T-BAC-*SNCA* mouse at 3 months of age demonstrates that a subset of large presynaptic terminals was immunopositive for phosphorylated α-synuclein. s: spine; T: presynaptic terminals with immunoreactivity for phosphorylated α-synuclein; t: presynaptic terminals lacking immunoreactivity for phosphorylated α-synuclein. Scale bars: 500 nm.

Last, we examined the precise localization of phosphorylated α-synuclein in striatal dendrites and spines in A53T-BAC-*SNCA* mice. A previous study (Taguchi et al., 2020a) using proteinase K-treated sections reported strong immunoreactivity for phosphorylated α-synuclein in the cortex, amygdala, and hippocampus of A53T-BAC-*SNCA* mice. Immunoreactivity in the striatum was relatively weak, but some punctate staining was observed. To further resolve the neuronal compartments that are immunoreactive for phosphorylated α-synuclein in A53T-BAC-*SNCA* mice, we used a pre-embedding immunogold EM labeling method. TEM observation demonstrated a selective localization of immunogold particles indicating that phosphorylated α-synuclein existed in a subset of presynaptic terminals (Fig. 6*A–E*). In addition, we observed a cluster of several immunogold particles in a number of instances. Furthermore, the immunoreactivity was only observed in a subset of large presynaptic terminals (Fig. 6*E*). No immunogold particles were observed in presynaptic terminals in sections from WT mice (data not shown).

## Discussion

Dendritic spines are vulnerable to structural changes during aging and in neurologic diseases (Ferrante et al., 1991; Dickstein et al., 2013; Mostany et al., 2013; Le et al., 2014; Pereira et al., 2014; Dorostkar et al., 2015; Herms and Dorostkar, 2016; Sato and Okabe, 2019). Previous studies have mainly used light microscopy or single-section TEM to assess structural changes in PD. However, light microscopy does not have sufficient resolution to pinpoint individual spines, and TEM images of single sections provide an incomplete picture of spine morphology, thus confounding our interpretation of ultrastructure. FIB/SEM is ideally suited to examine ultrastructure because of its ability to acquire 3D images of dendrites and spines in a completely automated manner, without the time-consuming preoccupations of image distortion, alignment, and section thickness variability that occur with serial section TEM. Furthermore, because the image acquisition is automated, simultaneous comparisons of neuropil can be performed among animal models across different ages in a reliable manner, with identical imaging conditions. To the best of our knowledge, this is the first study to systematically compare structural abnormalities across various ages in an animal model of PD.

We specifically chose the striatum for our study as this brain region is associated with both the prodromal and late stages of PD. Sleep disorder is one of the major hallmarks of prodromal PD, and the previous finding of abnormal expression of sleep-related gene networks in the striatum of PD patients (Jiang et al., 2019) indicates a possible link between this brain region and the prodromal stage of PD. Furthermore, the striatum is also known to be affected in the late phase of PD, when motor-related abnormalities become apparent. Thus, analysis of striatal neuropil can provide knowledge on the structural changes during PD both in the prodromal stage and as well as in the late phase.

An age-dependent decline in spine density was observed in both WT and A53T-BAC-*SNCA* mice. Studies have shown that dopamine facilitates dendritic spine formation and maintenance (Neely et al., 2007; Fasano et al., 2013; Dos Santos et al., 2017) and normal aging is associated with loss of dopamine producing cells in SNc and decrease in dopamine concentration in the striatum (Carlsson and Winblad, 1976; McGeer et al., 1977; Fearnley and Lees, 1991; Gibb and Lees, 1991). Furthermore, age-dependent decrease in the number of dopaminergic neurons in SNc has also been observed in A53T-BAC-*SNCA* mice (Taguchi et al., 2020a). Thus, it is highly likely that age-dependent decrease in spine density in both WT and A53T-BAC-*SNCA* mice depends on the level of dopamine in the basal ganglia. Supporting our view, the loss of dendritic spines in neurotoxin based animal model of PD was rescued by L-dopa treatment (Fieblinger et al., 2014; Suárez et al., 2014). It is also important to note that there were significantly fewer number of dopaminergic neurons in SNc of A53T-BAC-*SNCA* mice than in WT of corresponding ages (Taguchi et al., 2020a). This can have an important consequence in the development of PD pathology. Dopamine depletion in the striatum results in an increase in striatal neuron activity (Ohye et al., 1970; Arbuthnott, 1974; Schultz and Ungerstedt, 1978), and the excessive neuronal activity as a result of reduced dopamine content in A53T-BAC-*SNCA* mice could underlie the manifestation of PD.

Importantly, the decrease in spine density was accompanied by a concomitant increase in spine head size in the WT, but not mutant, mice. This result suggests that synaptic strength is conserved at all ages in WT mice. Similar to our finding, Calì et al. (2018) compared the synaptic surface area in layer 1 of the somatosensory cortex between 4- and 24-month-old mice and reported that the loss of spines at 24 months was balanced by an increase in spine head size, such that the total synaptic surface area remained constant between these two ages. A somewhat similar conclusion was also derived from previous studies of the prefrontal cortex (Duan et al., 2003; Dumitriu et al., 2010). Taken together with these earlier studies, our results support the emerging view that neurons tend to maintain an optimal excitation level and are endowed with homeostatic mechanisms to restore excitation thresholds to normal levels (Marder and Goaillard, 2006; Turrigiano, 2008). Furthermore, unlike in WT mice, the inverse relationship between spine head volume and spine density was not observed in A53T-BAC-*SNCA* mice, suggesting a disruption in the mechanisms that regulate homeostatic compensation of synaptic strength.

Which molecular mechanisms could play a role for the loss of age-dependent homeostatic enlargement of dendritic spines in A53T-BAC-*SNCA* mice? We argue that dopamine itself could be one of the major players for the bidirectional regulation of dendritic spine size. Dopamine is indispensable for induction of synaptic plasticity in striatal synapses (Reynolds et al., 2001; Gerdeman et al., 2002; Calabresi et al., 2007; Kreitzer and Malenka, 2007; Shen et al., 2008; Yagishita et al., 2014). The depleted state of dopamine in A53T-BAC-*SNCA* mice could result in an abnormal synaptic plasticity and misregulation of homeostatic balance in striatal synapse. In addition to the changes in dopamine levels, progressive accumulation of phosphorylated α-synuclein in the A53T-BAC-*SNCA* mice could also account for the loss of homeostatic enlargement of dendritic spines. Our pre-embedding immunogold labeling experiment showed accumulation of phosphorylated α-synuclein in the presynaptic terminals making synaptic contact with a subset of large mushroom-type spines. Because α-synuclein deposits inhibit the vesicular release of neurotransmitters from presynaptic terminals (Nemani et al., 2010; Scott et al., 2010), spines forming synaptic contacts with α-synuclein-positive terminals receive either sub-optimal presynaptic input or may even be completely devoid of glutamatergic signaling. Presynaptic activity is necessary for the survival of postsynaptic spines (McKinney, 2010); thus, the absence of vesicular release from the presynaptic terminal may render a subset of mushroom-type spines making synaptic contact with α-synuclein containing terminals non-functional and lead to their eventual elimination. This might explain the lower frequency of mushroom-type spines and smaller average head volume in the striatal spines of A53T-BAC-*SNCA* mice compared with WT mice. In turn, owing to the loss of a subset of mushroom-type spines, numerous small size spines may form as a homeostatic response to compensate for the reduction in total synaptic strength of dendrites in A53T-BAC-*SNCA* mice and result in higher spine density in A53T-BAC-*SNCA* mice than in the WT. The altered homeostatic regulation of dendritic spine size in A53T-BAC-*SNCA* mice is likely to cause a shift in the excitation-inhibition balance in the striatal network.

The lower frequency of mushroom-type spines in A53T-BAC-*SNCA* mice was also corroborated by a 2-fold decrease in the number of perforated spines in this PD mouse model. Thin-type spines primarily harbor macular, simple PSDs, while large, mushroom-type spines harbor perforated PSDs. Although the function of spine perforation remains unclear, studies have reported a correlative link between the abundance of perforated spines and cognitive ability in animals (Geinisman et al.,1986; Hara et al., 2012). Large, mushroom-type, perforated spines are believed to be the structural substrate for the storage of stable, non-malleable forms of memory; thus, the lower abundance of perforated spines in A53T-BAC-*SNCA* mice suggest a deficit in their long-term memory formation and storage.

Motor symptoms associated with PD in humans’ manifests later in life, typically after 50 years of age. However, in the present study, synaptic abnormalities in mice were already apparent at 1 month of age. It is crucial to mention that A53T-BAC-*SNCA* mice that we have used in this study recapitulate the symptoms and pathologies of prodromal PD (Taguchi et al., 2020a). Various non-motor related neurologic abnormalities associated with prodromal PD are already evident as early as two to three months of age in the mouse models that mimics prodromal PD (Paumier et al., 2013; Taguchi et al., 2020b), but the gross motor dysfunctions appear much later in life coinciding with the significant degeneration of dopaminergic neurons (Taguchi et al., 2020b). In A53T-BAC-*SNCA* mice, a significant decrease in the number of tyrosine hydroxylase (TH)-positive dopaminergic cells in the substantia nigra is observed at 9 and 18 months but not at 3 months (Taguchi et al., 2020a). This observation supports the prevailing idea that the onset of synaptic abnormalities and prodromal symptoms of PD precede the degeneration of dopaminergic cells and consequent manifestation of motor symptoms (Guidetti et al., 2001; Klapstein et al., 2001; Taguchi et al., 2020b). In future, it will be important to explore if distinct subcellular mechanisms are involved in the pathologies of the early prodromal stage and advanced, clinical stage of PD, and whether the former depends on the structural changes that occur at the synaptic level and the latter depends on the significant degeneration of dopaminergic cells in the basal ganglia.

Finally, it is noteworthy to mention that the structural observation of synapses at only one age provides an incomplete and misleading picture of the structural abnormalities during PD. Our study demonstrates that distinct structural changes occur at different ages in PD. We believe that the morphologies that we have characterized here are fundamental to the regulation of synaptic function and will serve as a basis for the physiological interpretation of synaptic abnormalities in PD.

## Synthesis

Reviewing Editor: Alexey Semyanov, Shemyakin-Ovchinnikov Institute of Bioorganic Chemistry Decisions are customarily a result of the Reviewing Editor and the peer reviewers coming together and discussing their recommendations until a consensus is reached. When revisions are invited, a fact-based synthesis statement explaining their decision and outlining what is needed to prepare a revision will be listed below. The following reviewer(s) agreed to reveal their identity: Hong-Yuan Chu, Igor Kraev.

Both reviewers have agreed that this is the first study in the field that systematically characterizes spine morphology changes during development in both health and a BAC mouse line carrying an a-synuclein mutation that causes Parkinson’s disease in humans. However, they have had a major concern that the sample is too small (n = 1 per group). You have taken multiple measurements from one animal and compared those to similar measurements from another animal. The results may simply reflect individual differences between the two animals. The reviewers believe that it is essential to increase the number of animals to 2-3 per group.

Please see the reviewers’ comments below.

Reviewer #1

In this manuscript, the authors applied FIB/SEM to characterize morphological changes of dendritic spines during development in both WT and BAC-A53T mice. The major findings of this study were the loss of homeostatic up-regulation of spine head volume during development in BAC-A53T mice, compared to WT mice, though mice in both groups exhibited age-dependent decrease in the dendritic spine density.

The study was well designed, and the findings were interesting and solid. I have a few comments for revision.

Major issues:

1. In neurotoxin-based animal models of parkinsonism, loss of striatal dendritic spines and synapses is Parkinson’s, and can be rescued by dopaminergic treatments, such as L-dopa (Fieblinger T et al, 2014). I would suggest the authors to discuss whether the decrease in the spine density in BAC-A53T (and WT) mice depends on changes in levels of dopamine in the striatum? For example, are there experimental evidence in literature showing an age-dependent decrease in the striatal dopamine levels in BAC-A53T mice?

2. Whether loss of homeostatic enlargement of spine heads in BAC-A53T mice are caused by increased alpha-synuclein level (Taguchi, et al, 2020), accumulation of pS129+ synuclein aggregates, or changes in dopamine levels. The quality of this manuscript would be greatly improved with further discussions of possible mechanisms.

3. Is it possible to do a correlation analysis between pS129+ immunoreactivity and the sizes/morphology of spine heads?

Minor issues:

1. It is not clear how the sample areas (15 cubic micrometer) were selected in the striatum, e.g. dorsal versus ventral striatum, or dorsaolaertal versus dorsomedial striatum. Given the difference in dopaminergic neuromodulation and/or biological function in movement control and motor learning of distinct striatal subregions, it is a critical piece of information to clarify to sample regions in the current study.

2. Page 14, L.9-10, please double check data of the spine neck length in 1- and 6-months groups. The median values and ranges do not match.

3. Page 32, L.15, miss labeling in the figure legend.

4. Figure 5 is not easy to read. I would suggest the authors to distinguish WT and BAC-A53T groups using different colors/symbols, or by explicitly labelling each panel.

Reviewer #2

The authors provided an interesting and extensive study on age dependent changes of dendritic spine morphology in the striatum and compared that development changes of the spines of alpha-synuclein BAC transgenic mouse model of Parkinson’s disease. Overall the results are of considerable interest. Main concern is about study size (n=1 per group).

Main points:

1. Sample size is too small (n=1 per group). It must be at least n=3 animals per group. It is not statistically correct to take multiple measurement from one animal and compare to similar measurements from another animal. The authors use FIB-SEM to obtain the data, which is labor intensive technique, probably that’s why they decided to use 1 animal per group. However, there are serial section TEM (more time consuming and tricky technique) studies that used sample size n>3 per group, so in current study is more than feasible to have at least 3 animal per group.

2. Immunolabelling shows phosphorylated α-synuclein in young (1 month) and old mutants. However Parkinson’s Disease (PD) develops in humans later in life. So, to compare young WT animals with young mutants and make some conclusions about PD is not totally correct. At least it must be discussed in discussion.

Minor points:

1. p.5 line 19. Immuno-labelling was done on mutants group only?

2. p.6 section ‘Anesthesia, fixation, and sectioning’. What was the speed of perfusion? Was it the same for morphology analysis and immuno?

3. p.6 line 10. PBS - please decipher because it wasn’t described anywhere in the paper before.

4. p.6 line 19. 1% uranyl acetate - is it aqueous or ethanol based?

5. p.7 line 10. FIB/SEM imaging - please state resolution in nm/px. I calculated it as 4.4nm/px, please correct me if I am wrong.

Image width of 13.8 μm means data set was actually ∼14um(X) x 9.2um (Y). Please state that. How many sections per dataset was obtained? Why 50nm increment was chosen?

6. p.7 line 22. Does it mean that a cube 1um length side was manually put next to spine neck and then the spine neck the length was roughly estimated? This has high possibility to be inaccurate. More precise measurement is needed.

How it was determined where the neck started and finished, where spine head started? On Fig.1, 6month animal dendrite - a neck of one of spines, I would argue, should have started closer to the dendrite.

7. p.7 line 24. Most of the dendrites are not fully visible in one image, so to measure its length one has to go through stack of images. How exactly the length of dendrite was measured?

Was dendrite diameter considered? Dendrites of different diameter could have different order and so have different spine density per length of dendrite.

Measuring spine density in volume of tissue could be one of good parameters to analyse density of spines (Aziz et al., 2019).

8. p.9 Statistics section. The authors should use two-way ANOVA to compare genotype and age.

9. p.9 lines 15-22. This paragraph is better placed into introduction or discussion.

10. p.10 line 4. Length of dendrite (mean, median or range) needs to be indicated.

11. p.11 line 24. Would suggest splitting spines into thin (small) and mushroom (big) spines and analyse their head size separately.

12. p.12 line 25. Same as previous comment. Suggesting to split spines into thin (small) and mushroom (big) spines and analyse their necks length separately.

13. p.14 line 5. Any particular reason why 0.04um3 was chosen? Needs to be explained.

14. p.14 line 6. Suggesting to use percentage instead of relative frequency. Having analysis of percentage of thin and mushroom spines would be better here.

15. Fig.3 A and B. Error bars for each point are needed.
